# Can Your Children Drive You To Drink? Stress and Parenting in Adults Interacting With Children With ADHD

**Published:** 1999

**Authors:** William E. Pelham, Alan R. Lang

**Affiliations:** William E. Pelham, Jr., Ph.D., is a professor in the Department of Psychology, State University of New York at Buffalo, Buffalo, New York. Alan R. Lang, Ph.D., is a professor in the Psychology Department, Florida State University, Tallahassee, Florida

**Keywords:** psychological stress, AOD (alcohol or other drug) consumption, parent, child, psychological AODC (causes of AOD use, abuse, and dependence), attention deficit disorder, behavioral problem, student, parenting skills, parent child relations, literature review

## Abstract

Several publications in the psychological literature support the theory that children are a major source of stress for their parents. Not surprisingly, parents of children with behavior problems—particularly children with attention deficit hyperactivity disorder (ADHD)—experience highly elevated levels of daily child-rearing stresses. Children with ADHD disregard parental requests, commands, and rules; fight with siblings; disturb neighbors; and have frequent negative encounters with schoolteachers and principals. Although many investigations have dealt with parenting stress caused by disruptive children, only a handful of studies have addressed the question of how parents cope with this stress. Those findings are presented, including a series of studies assessing parental distress and alcohol consumption among parents of normal children and ADHD children after the parents interacted with either normal- or deviant-behaving children. Those studies strongly support the assumption that the deviant child behaviors that represent major chronic interpersonal stressors for parents of ADHD children are associated with increased parental alcohol consumption. Studies also have demonstrated that parenting hassles may result in increased alcohol consumption in parents of “ normal” children. Given these findings, the stress associated with parenting and its influence on parental alcohol consumption should occupy a salient position among the variables that are examined in the study of stress and alcohol problems.

The idea that children can cause stress in parents is an often-exploited scenario in cartoon pages. “ Dennis the Menace” has tormented his parents and other adults for decades, and Calvin, the little boy in the cartoon series “ Calvin and Hobbes,” kept a record on his calendar of how often he drove his mother crazy. Similarly, in the noncartoon world, the question of whether children cause stress yields numerous raised hands in any group of parents. Indeed, a considerable number of publications in the psychological literature support the argument that children are a major source of stress for their parents (Crnic and Acevedo 1995).

Not surprisingly, parents of children with behavior problems—particularly children with attention deficit hyperactivity disorder (ADHD)—experience highly elevated levels of daily child-rearing stresses (Abidin 1990; Mash and Johnston 1990). Children with ADHD disregard parental requests, commands, and rules; fight with siblings; disturb neighbors; and have frequent negative encounters with schoolteachers and principals.

Although many investigations have dealt with parenting stress caused by disruptive children, only a handful of studies have addressed the question of how parents cope with this stress. For example, if stress in general can precipitate alcohol consumption, it would not be surprising to discover that some parents might attempt to cope with their parenting stress and distress by drinking. This article first reviews the relationship between childhood behavior problems and subsequent adult drinking behavior, and then explores the effects of child behavior on parental drinking. The discussion includes a review of a series of studies assessing parental distress and alcohol consumption among parents of normal children and ADHD children after the parents interacted with either normal- or deviant-behaving children.

## Childhood Behavior Disorders and Adult Alcohol Consumption

Children with ADHD have problems paying attention, controlling impulses, and modulating their activity level. Two other disruptive behavior disorders—oppositional defiant disorder (ODD) and conduct disorder (CD)—overlap considerably with ADHD. Children with ODD are irritable and actively defiant toward parents and teachers, whereas children with CD exhibit norm-violating behavior, including aggression, stealing, and property destruction. Substantial comorbidity occurs among these disorders, ranging from 50 to 75 percent. A large body of research has demonstrated many connections between alcohol problems in adults and these three disruptive behavior disorders (Pelham and Lang 1993):

- Children with externalizing disorders are at increased risk for developing alcohol or other drug (AOD) abuse and related problems as adolescents and as adults (Molina and Pelham 1999).
- Adult alcoholics more commonly have a history of ADHD symptomatology compared with non-alcoholics (e.g., Alterman et al. 1982).
- The prevalence of alcohol problems is higher among fathers of boys with ADHD and/or CD/ODD than among fathers of boys without these disorders (e.g., Biederman et al. 1990).
- Similarities exist between the behavioral, temperamental, and cognitive characteristics of many children of alcoholics and such characteristics of children with ADHD and related disruptive disorders (Pihl et al. 1990).

In summary, these findings indicate that childhood externalizing behavior disorders are associated with an increased risk of familial alcohol problems, as well as subsequent adult alcohol problems. Furthermore, parental alcohol problems may contribute to a child’s current and future psychopathology. Conversely, a child’s behavior problems may intensify parental drinking, which in turn may exacerbate the child’s pathology. This vicious cycle may result in ever more serious problems for the entire family.

## Effects of Childhood Behavior Problems on Parental Drinking

As described in the previous section, in families with children with behavior disorders and/or parental alcoholism, both the parents and children appear to have an elevated risk for alcohol-related problems. Researchers have only recently begun, however, to explore the causal mechanisms operating in these relationships. In addition, the research has focused primarily on the effects that parental drinking has on the children and their behavior. Some recent studies, however, have begun to examine the possible effects of deviant child behavior on parental alcohol problems.

Researchers and clinicians widely believe that children with behavior problems, particularly those with such externalizing disorders as ADHD, can adversely affect their parents’ mental health (Mash and Johnston 1990). Childhood externalizing problems frequently result in stressful family environments and life events affecting all family members, including parents. For example, numerous investigators have reported higher rates of current depression in mothers of children who were referred to a clinic because of behavioral problems than in mothers of healthy children (e.g., Fergusson et al. 1993). In addition, a significant correlation exists between daily parenting hassles (e.g., experiencing difficulty finding a baby sitter, having to talk to a child’s teacher, or coping with fighting among siblings) and child behavior problems. Thus, studies investigating the distressing effects of deviant child behavior on the immediate reactions and long-term functioning of parents have shown that exposure to difficult children is associated with dysfunctional parental responses, such as maladaptive discipline practices (Crnic and Acevedo 1995; Chamberlain and Patterson 1995).

**Figure 1:**
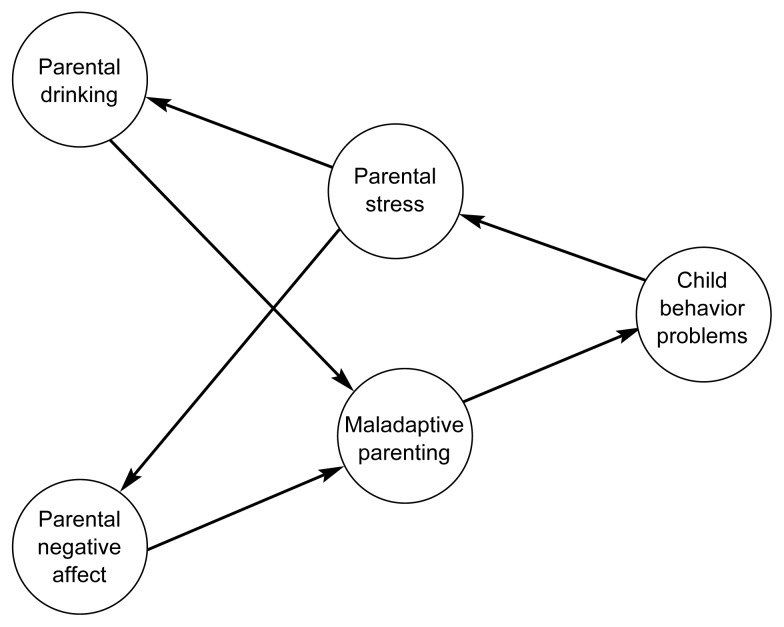
A model of the relationships among parental stress, child behavior problems, and parental drinking.

Despite the evidence that children with behavior problems cause substantial stress and other dysfunctional responses in their parents, almost no research has investigated whether these parental responses include elevated alcohol consumption and/or alcohol problems. This lack of research is particularly surprising given the well-documented association between adult alcohol problems and childhood externalizing disorders. Several relationships may exist among deviant child behavior, parental stress, and two broad types of dysfunctional responses in parents—emotional problems, such as anxiety and depression (i.e., negative affect), and problem drinking. These hypothesized relationships are shown in the model in figure 1, p. 294. The relationships among parental affect, drinking, and child behavior problems are believed to be transactional, with each variable influencing the other over time. In addition, various parental and child characteristics may influence these relationships. We have hypothesized that child behavior problems increase parental distress, which in turn influences drinking and parental affect. Drinking and negative affect result in maladaptive parenting behaviors, which exacerbate child behavior problems.

### Studies of the Influences of Child Behavior on Parental Drinking

Between 1985 and 1995, researchers at the University of Pittsburgh and Florida State University conducted a series of studies examining the relationships described above. Although some of those analyses have examined the influences of parental alcohol consumption on child behavior (Lang et al. 1999), most of the investigations have focused on the influences exerted by child behavior on parental behavior. Thus, these studies have manipulated child behavior and measured the resulting levels and changes in parental alcohol consumption. In order to determine the direction of effect in the documented associations between child behavior problems and parental drinking problems, the studies were conducted as experimental laboratory analogues, rather than as correlational studies in the natural environment.

Thus, all the studies described in this section have employed a similar design and similar measures. The participants, of whom most were parents and all were social drinkers (i.e., none were alcohol abstainers and none were self-reported problem drinkers), were recruited for what they believed were studies designed to investigate the effects of alcohol consumption on the way they interacted with children. The participants were told that they would have a baseline interaction with a child, followed by a period in which they could consume as much of their favored alcoholic beverage as they wanted (i.e., an ad lib drinking period), followed by another interaction with the same child. Each interaction period consisted of three phases: (1) a cooperative task in which the child and adult had to cooperate to solve a maze on an Etch-a-Sketch, (2) a parallel task during which the child worked on homework while the adult balanced a checkbook, and (3) a free-play and clean-up period. In all three settings, the adult was responsible for ensuring that the child stuck to the required task but also was directed to refrain from providing the child with too much assistance.

The adult participants were led to believe that the aim of the study was to compare their interactions with the children before and after drinking in order to learn about alcohol’s effects on adult-child interactions. The adults also were told that the child with whom they would interact might be a normal child from a local school or an ADHD child who was receiving treatment in a clinic. In fact, however, all of the children were normal children who had been hired and trained to enact carefully scripted roles that reflected either ADHD, non-compliant, or oppositional behavior (referred to as “deviant children” ) or normal child behavior (referred to as “normal children” ). The true goal of the study was to evaluate each adult’s emotional, physiological, and drinking behavior in response to his or her first interaction with a particular child and while anticipating a second interaction with the same child.

#### Studies Involving Undergraduate Students

Using undergraduate students as subjects, the first study of the series was designed to evaluate the validity of the concept that interactions with deviant children could induce both stress and stress-related alcohol consumption in adults (i.e., a proof-of-concept study) (Lang et al. 1989). In that study, both male and female subjects who interacted with deviant children reported considerably elevated levels of subjective distress and consumed significantly more alcohol compared with subjects who interacted with normal children. No significant differences in subjective distress or alcohol consumption existed between male and female subjects interacting with the deviant children. Thus, the study demonstrated that interactions with a deviant child could produce stress-induced drinking in young adults.

Intriguing as these results were, however, they could not be generalized to parents of children with behavior disorders, because the subjects were single undergraduate students who were not parents. The results did illustrate, however, that child behavior could be used to manipulate adult drinking behavior and that interactions with deviant children were potentially stressful, at least in young adults without parenting experience.

#### Studies Involving Parents of Normal Children

**Figure 2:**
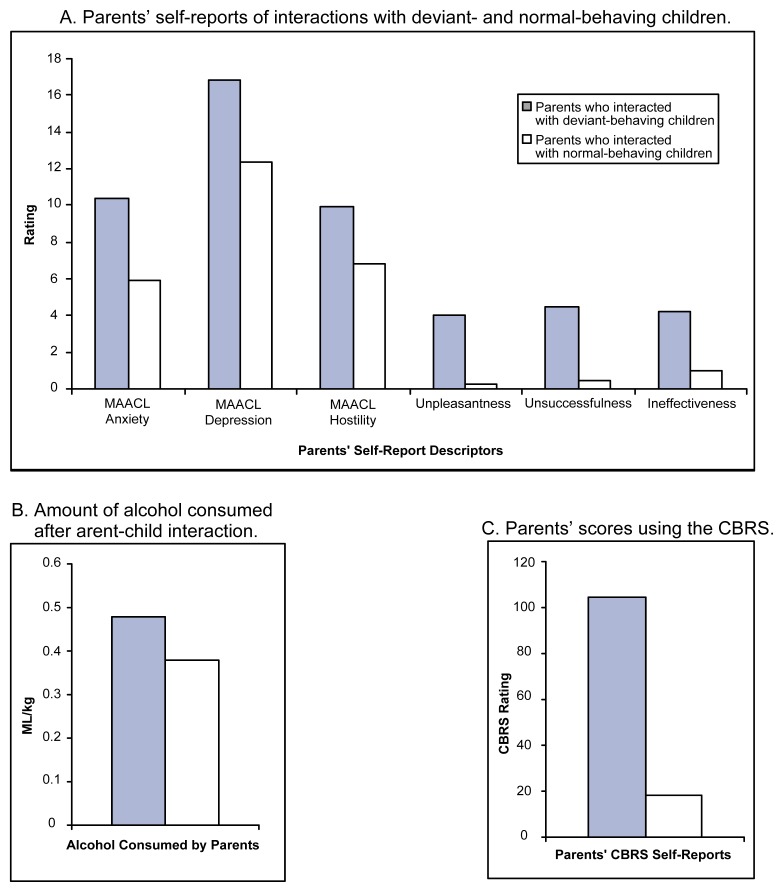
To measure the effects of children’s behavior on stress and alcohol use, parents of normal children interacted with boys who were trained to enact behaviors characteristic of either normal children or “deviant” children. Under these controlled experimental conditions, results showed that when exposed to deviant-behaving children, both mothers and fathers of normal children experienced considerable distress and consumed higher levels of alcohol compared with parents who interacted with normal-behaving children. (A) shows parents’ subjective self-reports of their interactions with deviant- or normal-behaving children. Higher bars reflect higher levels of distress, as interpreted by parents using the MAACL and single-item descriptors of the quality of the interaction. (B) shows the amount of pure alcohol (in milliliters per kilograms of bodyweight [mL/kg]) consumed by parents after interacting with either normal- or deviant-behaving children. Parents consumed more alcohol after interacting with deviant-behaving children. (C) shows parents’ total scores using the CBRS to describe their interactions with normal- and deviant-behaving children. Parents perceived much higher levels of misbe-havior in the deviant-behaving children. NOTE: Deviant children are defined as those who have behavioral problems, such as attention deficit hyperactivity disorder, oppositional defiant disorder, and conduct disorder. Normal children are defined as those children who do not meet the criteria for diagnosable psychopathology. MAACL = Multiple Affect Adjective Checklist. This checklist was used to ascertain the parents’ mood states. The list includes specific descriptors, such as anxiety, depression, and hostility, to describe the parent’s interactions with the children. CBRS = Child Behavior Rating Scale. Parents used this scale to describe children’s misbehavior (range = 0–140). Single-adjective measures = measures of each parent’s interpretation of the degree of pleasantness in interacting with the child [with 0 being very pleasant and 6 being unpleasant]; the parent’s success in getting the child to complete his tasks [with 0 being very successful and 6 being very unsuccessful]; and the parent’s view of his or her effectiveness in parenting that particular child [with 0 being very effective and 6 being very ineffective]). SOURCE: Pelham et al. 1997.

Using the same study design, Pelham and colleagues (1997) replicated these results with a sample of parents of normal children (i.e., children with no prior or current behavior problems or psychopathology). The subjects included married mothers and fathers as well as single mothers. The study found that both mothers and fathers were substantially distressed by interacting with deviant children and showed increases in negative affect and self-ratings of how unpleasant the interaction was overall, how unsuccessful they were in the interaction, and how ineffective they were in dealing with the child (see figure 2, p. 296). Moreover, parents from all three groups who interacted with a deviant child consumed more alcohol than did parents who interacted with a normal child. Interestingly, for both reported subjective distress and drinking behavior, the differences between subjects interacting with deviant and normal children were considerably larger among parents of normal children than among college students in the investigation by Lang and colleagues (1989). These findings indicate that when parents are presented with a stress-inducing factor (i.e., an ecologically valid stressor) relevant to their normal life, such as child misbehavior that induces considerable subjective distress, they may engage in increased alcohol consumption (i.e., stress-induced drinking).

It is notable that these effects were obtained in a sample of parents of non-deviant children. Thus, the results are consistent with other studies showing that parenting hassles can cause distress even in normal families (Crnic and Acevedo 1995; Bugental and Cortez 1988). Furthermore, because the effects were obtained in both mothers and fathers, the study demonstrated that problematic child behavior can influence drinking behavior regardless of parent gender. Among the mothers studied, interactions with deviant children had the largest impact on single mothers, who have also been shown to be particularly vulnerable to numerous stressors, including parenting difficulties (Weinraub and Wolf 1983) and drinking problems (Wilsnack and Wilsnack 1993).

#### Studies Involving Parents of ADHD Children

To explore the link between alcohol problems and deviant child behavior in parents of children with ADHD, Pelham and colleagues (1998) employed the same study design with a sample of parents who had children with an externalizing disorder. Again, the study included single mothers as well as married mothers and fathers to allow analysis of potential differences in drinking behavior as a function of gender and marital status. In addition, after the initial data analysis, the investigators conducted an unplanned analysis using the Michigan Alcoholism Screening Test to determine problematic drinking behavior of the subjects’ parents and associated familial risk for drinking problems. This analysis was prompted by considerable research indicating that familial history of alcohol problems may be associated with the effects of stress and alcohol on a person’s behavior (Cloninger 1987).

As in the studies by Lang and colleagues (1989) and Pelham and colleagues (1997), parents of ADHD children responded with self-ratings of increased distress and negative affect after interactions with the deviant children. The magnitude of the elevations in parent distress was as great as that seen in parents of normal children. Because parents of children with disruptive behavior disorders are exposed to such deviant child behavior on a daily basis, these observations suggest that those parents experience chronic interpersonal stressors. Other studies have indicated that such chronic interpersonal stressors have a greater impact in causing negative mood states (e.g., depression) in adults than do onetime (i.e., acute) and/or non-interpersonal stressors (Crnic and Acevedo 1995). Consequently, these findings illustrate the importance of child behavior on parental stress and mood levels.

Despite the increased distress levels, however, parents of ADHD children as a group did not display the stress-induced drinking shown by college students or parents of normal children. Deviant child behavior resulted in elevated drinking levels only when the investigators conducted the subgroup analyses based on family history of alcohol problems. Thus, parents with a positive family history of alcohol problems exhibited higher drinking levels after interacting with deviant children than after interacting with normal children. Conversely, parents without a family history of alcohol problems showed lower drinking levels after interacting with deviant children than after interacting with normal children.

This finding was somewhat surprising, because the investigators had strongly expected parents of ADHD children as a group to exhibit elevated drinking in response to deviant child behavior. The study results suggest, however, that some parents of ADHD children (i.e., parents without a family history of alcohol problems) may have developed coping techniques other than drinking (e.g., reducing their alcohol consumption or establishing problem-solving strategies) to cope with the stressors associated with raising a child with deviant behavior. Consequently, it is important to measure additional differences among individuals in order to fully explain responses to various types of child behavior.

Notably, the effect of a family history of alcohol problems on drinking levels was comparable for mothers and fathers. Most previous studies had demonstrated an association between a positive family history and alcohol problems in men, whereas the evidence for such an association in women was less convincing (Gomberg 1993). Furthermore, two distinct subgroups of parents, differentiated by their family history of alcoholism, appeared to exist, and they exhibited different coping techniques. Thus, parents with a family history of alcohol problems more commonly used maladaptive, emotion-focused coping techniques (i.e., drinking), whereas parents without such a history more commonly used adaptive, problem-focused coping techniques (i.e., not drinking). Accordingly, the researchers continued to explore whether these subgroups also existed among mothers of ADHD children.

To facilitate data interpretation, the investigators modified the study design in several ways, as follows:

- They determined the subjects’ family histories of alcohol problems, defined as having a father with alcohol problems, prior to the study and used this information as a criterion for subject selection.
- They quantified stress-induced drinking for each subject using a within-subject design rather than the between-subject design employed in previous investigations. Thus, rather than comparing subjects who had interacted with a deviant child with subjects who had interacted with a normal child, the investigators had each subject participate in two laboratory sessions 1 week apart. In one session, the subject interacted with a deviant child and in the other session she interacted with a normal child.
- They measured the subjects’ heart rate and blood pressure during their interactions with the children in order to obtain physiological information about subjects’ stress levels.
- They administered numerous tests in order to identify dispositional characteristics, such as psychopathol-ogy, personality, coping, attributional style, alcohol expectancies, life events, family functioning, and drinking history, which might influence the subjects’ response in addition to the family history of alcohol problems.

The results of the study confirmed the previous findings on the effects of child behavior on parental stress levels that were obtained from college students and parents of normal children. After interacting with the deviant children, the mothers of ADHD children showed greater physiological distress (i.e., significantly increased heart rate and blood pressure) than after interacting with the normal children. These mothers also showed greater subjective distress (i.e., increased negative affect; decreased positive affect; and increased self-ratings of unpleasantness, unsuccessfulness, and ineffectiveness). Furthermore, the mothers consumed approximately 20 percent more alcohol after interacting with the deviant children than after interacting with the normal children (Pelham et al. 1996*a*).

These findings clearly demonstrate that interactions with ADHD children engender large stress responses from their mothers in multiple domains. Furthermore, the mothers in this study as a group coped with this distress by drinking more alcohol. Contrary to the family history analysis in the previous study (Pelham et al. 1998), however, the subject’s paternal history of alcohol problems (selected in advance) did not affect alcohol consumption in this larger sample.

To further clarify the results of the study among mothers of ADHD children, the researchers also evaluated the mothers’ dispositional characteristics before their interactions with the children to identify potential associations with their stress-induced drinking (Pelham et al. 1996*b*). The investigators correlated these measures with the amount of alcohol the mothers consumed after interacting with a deviant child (i.e., stress-induced drinking), controlling for the amount of alcohol consumed after the interaction with the normal child. These analyses identified numerous factors associated with higher levels of stress-induced drinking, including the following:

- Higher levels of routine drinking (i.e., a greater number of drinks per drinking occasion)
- More negative consequences of drinking
- Higher levels of drinking problems
- A denser family history of alcohol problems (i.e., alcoholic relatives in addition to the father)
- Maternal history of drinking problems
- Higher self-ratings of using mal-adaptive coping strategies, feeling depressed, and experiencing more daily life stressors.

Although many mothers of ADHD children showed elevated drinking levels in response to interacting with a deviant child, a substantial number of mothers decreased their alcohol consumption after such interactions. This pattern of divergent responses is comparable to the one observed among mothers of ADHD children in the earlier study by Pelham and colleagues (1998) and points to the need for more fine-grained analysis.

The individual differences in coping with deviant child behavior noted in both studies suggest that alcohol consumption in mothers of ADHD children is a complex phenomenon. Clearly, some mothers resort to maladaptive coping mechanisms (i.e., drinking) in response to the stress of dealing with their child. Such a dysfunctional coping response often can be predicted by the mothers’ general coping styles. Other mothers, however, cope in a problem-solving fashion by decreasing their alcohol consumption when anticipating another interaction with the deviant child, apparently believing that drinking would decrease their effectiveness in interacting with that child.

Whereas a paternal history of alcohol problems did not predict stress-induced drinking in the mothers of ADHD children, a maternal history of alcohol problems and the frequency of alcohol problems in other first-degree relatives did predict stress-induced drinking. These findings suggest that in addition to, or instead of, paternal alcohol problems, researchers should consider maternal drinking history and family density of drinking when assessing the influence of family history on female drinking behavior.

The study on the mothers of ADHD children, as well as all the other studies in this series, was conducted in an “artificial” laboratory setting. The fact that subjects’ self-reported drinking levels (i.e., number of drinks per occasion) and self-reported alcohol problems correlated highly with stress-induced drinking measured in this setting confirms that this type of investigation can generate information that reflects real-life behavior.^1^ Thus, the laboratory findings provide strong support for the hypothesis that among mothers of ADHD children, routine drinking and drinking problems are at least in part a response to the daily stress of coping with their children.

## Conclusions

A recent review of the relationship between AOD abuse and parenting concluded that huge gaps exist in understanding the association between parental alcohol abuse and parent-child relationships (Mayes 1995). For example, more information is needed regarding the effects of alcohol on parenting behaviors (e.g., overly punitive discipline) that are known to affect child development. Lang and colleagues (1999) recently demonstrated in a laboratory setting that alcohol negatively influences parenting behaviors (e.g., lax monitoring) that mediate the development of conduct problems in children (Chamberlain and Patterson 1995). This finding confirms the parent-to-child influence on the relationship between parental alcohol problems and externalizing behavior problems in children. Conversely, the studies described in this article strongly support the assumption that the deviant child behaviors that represent major chronic interpersonal stressors for parents of ADHD children (Crnic and Acevedo 1995) are associated with increased parental alcohol consumption, thereby confirming a child- to-parent influence on the same relationship.

Childhood externalizing disorders affect approximately 7.5 to 10 percent of all children, with a considerably higher incidence among boys. The association between childhood behavior disorders and parental alcohol problems means that many adults with drinking problems are parents of children with behavior problems. Moreover, the study by Pelham and colleagues (1997) involving parents of normal children has demonstrated that parenting hassles may result in increased alcohol consumption even in normal families. Together, the results described in this article indicate that the stress associated with parenting and its influence on parental alcohol consumption should occupy a salient position among the variables that are examined in the study of stress and alcohol problems.

## Footnotes

1 Some of the subjects reported alcohol problems even though the sample only included women classified as
